# Program of All-Inclusive Care for the Elderly: an untapped setting for research to advance pain care in older persons

**DOI:** 10.3389/fpain.2024.1347473

**Published:** 2024-04-22

**Authors:** Catherine Riffin, Lauren Mei, Lilla Brody, Keela Herr, Karl A. Pillemer, M. Carrington Reid

**Affiliations:** ^1^ Department of Medicine, Weill Cornell Medicine, New York, NY, United States; ^2^ College of Nursing, University of Iowa, Iowa City, IA, United States; ^3^ College of Human Ecology, Cornell University, Ithaca, NY, United States

**Keywords:** older adults, pain management, chronic pain, evidence-based intervention, Program of All-Inclusive Care for the Elderly

## Abstract

The Program of All-Inclusive Care for the Elderly (PACE) is a community-based care model in the United States that provides comprehensive health and social services to frail, nursing home-eligible adults aged 55 years and older. PACE organizations aim to support adequate pain control in their participants, yet few evidence-based pain interventions have been adopted or integrated into this setting. This article provides a roadmap for researchers who are interested in collaborating with PACE organizations to embed and evaluate evidence-based pain tools and interventions. We situate our discussion within the Consolidated Framework for Implementation Research (CFIR), a meta-theoretical framework that considers multi-level influences to implementation and evaluation of evidence-based programs. Within each CFIR domain, we identify key factors informed by our own work that merit consideration by research teams and PACE collaborators. Inner setting components pertain to the organizational culture of each PACE organization, the type and quality of electronic health record data, and availability of staff to assist with data abstraction. Outer setting components include external policies and regulations by the National PACE Association and audits conducted by the Centers for Medicare and Medicaid Services, which have implications for research participant recruitment and enrollment. Individual-level characteristics of PACE organization leaders include their receptivity toward new innovations and perceived ability to implement them. Forming and sustaining research-PACE partnerships to deliver evidence-based pain interventions pain will require attention to multi-level factors that may influence future uptake and provides a way to improve the health and well-being of patients served by these programs.

## 1 Introduction

Chronic pain is a major public health issue with profound societal and economic impacts. In the United States, estimated costs attributable to pain exceed $296 billion annually (1). Globally, pain is the leading cause of disability (2–4). It is also one of the most common reasons adults seek medical care (5). Inadequately managed pain negatively affects multiple aspects of health and well-being, including cognitive processes, mood and mental health, sleep, social interactions, and overall quality of life (6, 7). Critically, pain may accelerate functional decline, contribute to reductions in physical activity, and increase mortality risk (8).

Chronic pain is particularly problematic in older adults (>65 years). According to recent estimates, more than half of U.S. older adults experience bothersome pain (1, 9). Pain in later life is often inadequately managed. Pain treatment in older adults is complicated by a range of factors, including altered drug absorption, frequent co-occurrence of nociceptive and neuropathic pain, and older adults’ own attitudes and beliefs about pain treatments (6). Moreover, pharmacologic treatment of older adults' pain is commonly associated with adverse side effects such as urinary retention, constipation, bleeding, and risk of falls. For persons with dementia, side effects may also include respiratory depression and delirium (10). Thus, implementation of best practices for pharmacological intervention for pain management are essential in settings in which vulnerable older adults reside.

Recognition of these issues alongside the rising costs of pain care and opioid crisis have prompted federal and state agencies, including the National Institute on Aging (NIA) and Centers for Disease Control (CDC), to invest in the development and evaluation of nonpharmacologic pain treatments that can address the multidimensional experience of pain (11, 12). Nonpharmacologic interventions involve physical and psychological strategies to alleviate pain and discomfort and may be delivered alone or in conjunction with pharmacologic approaches. In randomized trials, nonpharmacologic interventions, encompassing acupuncture, massage, music therapy, reflexology, and cognitive behavioral therapy have been shown to be safe and effective for older adults (10, 13, 14). Despite these benefits, the transfer of empirically supported nonpharmacologic interventions into real-world practice lags behind the evidence base (15). For healthcare to meet the needs of the 51.6 million U.S. adults with pain conditions (16), broad dissemination and implementation of these evidence-based interventions is paramount.

The Program of All-Inclusive Care for the Elderly (PACE) represents a significant public health opportunity to support effective pain assessment and management in medically complex, frail older adults. PACE delivers comprehensive health and social services to low-income individuals (aged 55 years and older) who are eligible for nursing home admission but choose to receive long-term care in community settings. Originally developed to address the long-term care needs of older immigrants in San Francisco, California, PACE now operates in 32 states and the District of Columbia. At present, there exist 154 PACE programs across the United States serving more than 70,000 participants. PACE therefore affords a unique opportunity to embed and evaluate pain interventions in older adults who are differentially affected by social determinants of health.

Under a capitated financing arrangement funded by Medicare and Medicaid, PACE services are coordinated and delivered by an interdisciplinary team of health professionals comprising physicians, nurses, physical therapists, occupational therapists, social workers, and others. This interdisciplinary approach aligns with national task force recommendations for collaborative, team-based pain care that addresses the multidimensional nature of pain through a combination of physical, psychological, and behavioral interventions (11, 15). As PACE aims to prevent functional decline that may necessitate costly hospitalizations and nursing home placement among PACE participants (17), addressing pain as a major risk factor for functional decline is a crucial imperative. However, because few evidence-based pain assessment tools and interventions have been adopted in PACE organizations, consideration of implementation factors and processes is essential. The integration and uptake of evidence-based pain tools and programs within PACE organizations will require careful attention to multiple factors and processes that may affect research translation. The Consolidated Framework for Implementation Research (CFIR) is a meta-theoretical framework that considers multi-level influences to implementation and evaluation of evidence-based programs, including characteristics of the outer setting (external demands, pressures, and policies), inner setting (features attributable to individual PACE organizations), individuals (personal attributes of PACE leaders and staff) and processes (quality of planning and engagement of relevant stakeholders) (18). CFIR's theory-based constructs and mechanisms can be used as a lens to identify factors that may influence whether an intervention is adopted or not as well as potential barriers and facilitators to implementation.

Using CFIR as a guiding framework, this paper provides a roadmap for researchers who are interested in collaborating with PACE organizations to embed and evaluate pain interventions that are evidence-based. We draw on examples from our work involving a multi-site clinical trial to discuss practical considerations for initiating and sustaining research-PACE partnerships that would apply to other evidence-based interventions. In brief, our multi-site trial aims to evaluate a caregiver-targeted training program to improve pain assessment among persons with dementia (19), which is a crucial element in the treatment of pain. The intervention is delivered over four weekly telephone sessions (30–60 min each) by a trained interventionist and provides caregiver training in observational pain assessment, coaching in effective pain communication, and structured opportunities for skill-building. Caregivers who are not randomized to the intervention condition participate in an attention control condition that parallels the delivery and time commitment of the intervention, but focuses on health promotion topics, such as nutrition, exercise, and sleep. We began by piloting the intervention in one PACE organization in 2022. We subsequently initiated collaborations with five other PACE programs across the United States in preparation for the multi-site trial. In the sections that follow, we situate the lessons learned from our experience within the CFIR framework, focusing on characteristics of the outer setting, inner setting, and individuals. Our core recommendations are summarized in Table 1 and described in detail below.

**Table 1:** Recommendations for collaborating with PACE organizations.

CFIR construct	Recommendation
Outer setting characteristics	
- External pressures and policies - Financing - Partnerships	- Initiate early discussion about PACE reporting requirements and timeframes, including CMS audits and state-level reports. - Discuss the allocation of potential funding and resources. - Consider how the evidence-based program will be sustained after the conclusion of the funding period.
Inner setting characteristics	
- Technology infrastructure - Work infrastructure - Culture	- Collaborate with PACE program administrators to identify data and measures that are available within the EHR. - Embed recruitment procedures within existing workflows. - Initiate conversations to understand the organization's values, beliefs, and norms around using data to inform practice.
Individual characteristics	
- High-level leaders and key decision-makers - Implementation facilitators - Implementation team members - Planning and tailoring	- Develop simple communication materials to present to high-level decision makers; include value propositions and potential benefits to PACE participants. - Identify PACE program staff (EHR administrators; members of the IDT) who will assist with specific research activities. - Establish regular meetings to track and ensure progress; include multiple contacts to mitigate risks of staff turnover. - Allow time to co-design and tailor recruitment materials to each PACE organization's membership.

## 2 Features of the outer setting

Outer setting constructs pertain to external influences—regulations, policies, and financing—that may influence an intervention's implementation. At the national level, PACE operates under a regulatory framework in which organizations require licensure to deliver medical care in clinic settings and coordinated services (encompassing social, behavioral, and health care) in the home. All PACE organizations must be approved by the Centers for Medicare and Medicaid Services (CMS) and comply with quality standards and reporting (20). CMS conducts regular audits to monitor the care quality and health outcomes of PACE participants and to inform quality improvement initiatives. PACE organizations are also subject to quality monitoring and reporting at the state level (21, 22). To support the continued advancement of PACE quality and growth, the Alliance for PACE Innovation and Quality (APIQ), sponsored by the National PACE Association (NPA) (23) provides consultation to individual PACE organizations.

Awareness of CMS and NPA requirements and timeframes is essential for research teams, given the implications for participant recruitment and enrollment. CMS audits, for example, are a lengthy process. The initial phase is a six-week period in which the PACE organization is notified of its selection and required to prepare and submit data reports (24). This phase is followed by additional field work by CMS. Once the final audit report is issued, the PACE organization must design and implement corrective action plans for each critique. Responses must be submitted within specified timeframes (e.g., 30 days to submit corrective action plans). Program audits require significant time and effort from PACE staff, thus limiting their capacity to take on new projects or engage in research activities.

Our research team worked closely with our PACE collaborators to modify the research timeline to accommodate real-world practice constraints. Initiating such discussions early in the partnership also helped us to identify alignment between our primary outcomes of interest and measures that were being collected by our PACE partner as part of their routine reporting. In New York State, for example, value-based payment quality measures include the percentage of PACE participants who (a) did not have an emergency room visit in the last 90 days, (b) remained stable or demonstrated improvement in (i) pain intensity and (ii) Nursing Home Level of Care (NFLOC) score, and (c) did not experience uncontrolled pain (25). Understanding PACE reporting requirements at the state and national levels was foundational to our team's ability to collect data that was relevant to our study goals and feasible to extract in the context of existing PACE workflows. Such discussions may be particularly helpful for research teams conducting embedded pragmatic trials that require the use of existing administrative data (26, 27).

Beyond regulatory standards and reporting, an additional outer setting construct relates to financing. As noted, PACE services are delivered through a capitated payment structure. In contrast with fee-for-service plans, capitation allows PACE providers to be flexible in the services they deliver, offering comprehensive, preventative care that is tailored to individuals' needs. This model is well aligned with the uptake and integration of evidence-based pain interventions, especially those that have a strong case for potential cost-savings, for example, in the form of averted hospitalizations or delayed institutionalization.

Apart from PACE's financing, research teams with access to external resources (e.g., funding from federal grants) should discuss the potential allocation of funds at the outset of the collaboration; for example, whether the research team will compensate PACE personnel for time spent on research activities or provide the PACE program with a stipend or honorarium for their engagement in the project. Conversations about funding should also consider how the evidence-based intervention will be sustained once the funding period ends. We explicitly discussed how members of the PACE interdisciplinary team could eventually be trained in administering our intervention and how the program could be covered under existing reimbursement structures.

## 3 Features of the inner setting

Within the inner setting, CFIR constructs encompass structural and cultural characteristics of the organization. Two primary components are information technology and work infrastructure. PACE organizations vary considerably with respect to the type and quality of electronic health record (EHR) data they collect as well as the availability of program staff to assist with data abstraction. As PACE programs are operated independently, there is no standardized set of measures that all programs collect within their EHR. For example, some PACE programs use the Functional Assessment Staging Tool (FAST) to evaluate participants' physical function, others use NFLOC as a marker of functional impairment, and others do not have a standardized measure of function. Participants' pain levels and treatments are also not systematically recorded. The heterogeneity in EHR documentation is partially due to the lack of uniformity in EHR systems across PACE organizations. While most programs use Epic, PointClicCare, or Netsmart; others use TruChart/Mediture or NextGen (28).

The variability in EHR data collection and documentation across PACE organizations poses challenges for multisite trials that require standard measures for outcome and process evaluations. We collaborated with PACE program administrators and analysts to identify data elements that are available for abstraction as well as those that could be included for research purposes (e.g., simple pain scales, such as a pain thermometer). We also considered how data abstraction would be performed: by PACE program staff or by a member of our research team. PACE program staff are well situated to conduct the data abstraction given their intimate knowledge of the EHR system but may have limited time beyond their daily activities and may experience turnover, which can compromise longitudinal data abstraction from multi-year studies. Research personnel may have greater capacity to perform the data abstraction but require start-up investments, such as training by PACE staff in medical chart review, onboarding by the PACE organization to enable EHR access (e.g., credentials as a PACE volunteer), and completing relevant documentation for accessing the data; for example, Data Use Agreements (DUA) and Institutional Review Board (IRB) reliance agreements. As most PACE organizations do not have their own IRBs, research teams will need to work with their home institutions to execute IRB agreements to ensure participant privacy and data safety.

CFIR also identifies organizational culture as a construct that may impede or facilitate implementation of evidence-based programs. Careful consideration should be given to each PACE organizations’ values, beliefs and norms around integrating novel tools and interventions. In our experience, PACE programs are highly receptive to integrating evidence-based interventions, given the alignment between pain intervention outcomes and PACE's guiding mission to help individuals maintain independence and functioning. However, most programs need to evaluate the priority and timing of new projects against other quality improvement initiatives and care delivery innovations. In some cases, we delayed the onset of participant recruitment to accommodate a PACE organization's ongoing initiatives. This was a useful strategy for ensuring maximum attention and participation among PACE organization leaders and staff.

## 4 Individual-Level characteristics

**Figure 1:**
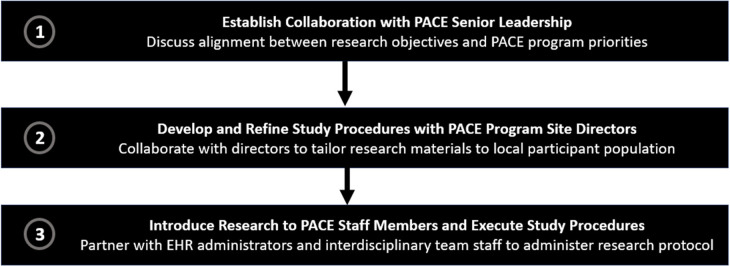
Steps for engaging PACE decision-makers and other stakeholders in research collaborations.

At the individual level, CFIR identifies several types of decision-makers that may influence an intervention's uptake, including individuals with high levels of authority and persons who are ultimately responsible for implementation. An overview of the steps we took to engage PACE decision-makers and other key stakeholders is presented in Figure 1.

A crucial first step in our own work was to approach high-level executives with value propositions that describe how and why the intervention is aligned with the PACE program's priorities and its relevance to their participants (29). In our case, we highlighted the potential links between effective pain control in persons with dementia and unnecessary hospitalization and premature nursing home placement, as these outcomes were key quality measures for our PACE partners. We developed structured communication materials, including an executive summary and slide deck that described the aims, objectives, and potential impact of our intervention, emphasizing the study's potential to improve PACE's pain practices and ultimately impact important quality measure outcomes (e.g., pain intensity and control, emergency room visits). During these initial meetings, we sought to understand each PACE program's priorities for their participants and organization, with the goal of identifying potential barriers and facilitators to collaboration. Although we expected that outer setting (e.g., CMS audits) and inner setting (e.g., EHR data) characteristics would be the most influential factors in determining whether the program would agree to collaborate, we found that PACE executives who either had personal or clinical experience caring for someone with pain and dementia were most receptive to our research. In view of their experience, these individuals were willing to discuss strategies for overcoming logistical challenges, particularly those within their control at the inner setting (e.g., EHR data abstraction, availability of personnel) to implement the intervention.

Whereas PACE organization executives were influential in determining whether the research partnership was possible, PACE program staff were instrumental in operationalizing the study procedures. EHR administrators, for example, assumed responsibility for generating relevant data reports and training our own research staff in data abstraction. Interdisciplinary team members played a key role in participant recruitment and enrollment, facilitating “warm hand-offs” between potential research participants and our own study team. Despite the strong alliances we formed with PACE staff members, an unexpected challenge was high staff turn-over. As such, having multiple points of contact at each PACE organization was essential to ensuring continued progress and accurate longitudinal data abstraction. Further, an established meeting structure with regular touch points was key to maintaining progress among task-oriented groups (e.g., teams responsible for participant recruitment and data abstraction).

An additional strategy at the individual level was to harness PACE site directors' knowledge of their membership to tailor intervention materials to the local context. As PACE organizations are located in diverse geographic regions across the United States, each program serves a unique population with different cultural backgrounds and languages spoken. PACE site directors offered important insights about their participants' characteristics and guidance for tailoring our study recruitment materials, often offering to print the recruitment materials on their letterhead to enhance receptivity among their participants. Overall, forging close partnerships with site directors and staff served to enhance the relevance of our work to PACE participants.

## 5 Discussion

The scale and spread of evidence-based pain assessment and communication tools and treatments is fundamental to supporting adequate pain control in our aging population. PACE programs represent an important opportunity to embed and disseminate evidence-based interventions to improve the care quality and outcomes of older persons with chronic pain. Currently, there exists no clear roadmap for forging research-PACE partnerships to embed evidence-based programs. This paper takes an initial step toward that goal by highlighting examples from our own work and situating them within an implementation science framework, CFIR.

With respect to outer setting characteristics, national- and state-level requirements impacted the timing and scope of our collaborations. We recommend that research groups initiate discussions with PACE organizations early in the research process to consider how the research will be carried out in the context of real-world pressures, focusing on potential synergies—for example, potential overlap between PACE reporting requirements and research outcomes—rather than roadblocks. Further consideration should be given to financing arrangements, such as grant funding and external resources from the research team, that may be deployed during the project period, as well as strategies for ensuring the intervention's sustainability at the conclusion of the research timeline.

At the inner setting, we recommend that research teams work closely with PACE program administrators to identify data and measures that are already available within each EHR system and discuss how recruitment procedures can be embedded within existing workflows. At the individual level, a fruitful strategy was presenting PACE executives with a high-level summary of the project that underscored how the intervention would improve their pain practices and quality measure outcomes. Once a program agrees to collaborate, initial conversations should delineate how specific tasks will be performed and by whom (e.g., who will conduct EHR data abstraction, facilitate participant recruitment). Subsequent meetings should have a clear structure and cadence (e.g., bi-weekly 30-min case reviews with troubleshooting) and engage multiple points of contact. Overall, consistent open dialogue with PACE partners was fundamental to ensuring the launch and maintenance of our collaboration.

## 6 Conclusion

The future of health care delivery has reached an inflection point as unprecedented numbers of older adults are living longer with debilitating pain conditions that limit their functioning and quality of life. While adequate pain control for our aging population is a public health imperative, evidence-based assessment approaches and pharmacologic and nonpharmacological strategies are under- or ineffectively utilized. PACE organizations are well situated to facilitate the uptake and dissemination of pain treatments given their capitated finance structure and interdisciplinary approach to care delivery. Thus, investing in research-PACE partnerships is crucial to ensuring the translation of proven pain interventions and ultimately improving pain control in older adults.

## Data Availability

The original contributions presented in the study are included in the article, further inquiries can be directed to the corresponding author.
